# The crucial value of serum ferritin in assessing high-risk factors and prognosis for patients with endometrial carcinoma

**DOI:** 10.1186/s12905-023-02575-x

**Published:** 2023-08-07

**Authors:** Jiali Liu, Beibei Xuan, Quan Quan, Sainan Gong, Xiaoling Mu

**Affiliations:** Department of Gynecology, The First Affiliated Hospital of Chongqing Medical University, No. 1 Youyi Road, Yuanjiagang, Yuzhong District 400016 Chongqing, China

**Keywords:** Endometrial carcinoma, Serum ferritin, Inflammation, Ferroptosis

## Abstract

**Background:**

Endometrial carcinoma is a common malignant tumor in female reproductive system. At present, there is no effective and economic prognostic index. This study aimed to investigate the effect of serum ferritin levels on the prognosis of endometrial carcinoma.

**Methods:**

Data of 367 patients who diagnosed with endometrial carcinoma at the First Affiliated Hospital of Chongqing Medical University between January 2012 and August 2018 was retrospectively analyzed. The prediction accuracy was evaluated by receiver operating characteristics curves and Youden's J statistics. Hosmer–lemeshow test was used to confirm the goodness of fit of the model. The prognostic value of serum ferritin on disease free survival (DFS) and overall survival (OS) of endometrial carcinoma was evaluated by univariate log-rank tests and multivariate cox regression models.

**Results:**

Preoperative high serum ferritin was correlated with older age, high grade, specific histological subtypes and recurrence of endometrial carcinoma (*P* < 0.05). The DFS and OS of 198 patients with elevated serum ferritin levels were significantly lower than those with low serum ferritin levels (*P* = 0.001 and *P* = 0.002, respectively). In multivariate analysis, serum ferritin was an independent prognostic factor for DFS and OS in endometrial carcinoma (*P* = 0.012, *P* = 0.028).

**Conclusion:**

Through our research, we found that the high expression of serum ferritin level was not only related to low DFS and OS in patients with endometrial carcinoma, but also related to the high-risk factors of endometrial carcinoma recurrence. So serum ferritin levels may be used to predict the poor prognosis of patients with endometrial carcinoma.

**Supplementary Information:**

The online version contains supplementary material available at 10.1186/s12905-023-02575-x.

## Introduction

Endometrial carcinoma (EC) is a group of epithelial malignant tumors that often occur in perimenopausal and postmenopausal women. With the increasing obesity rate of modern women [1], its incidence rate and mortality rate have been increasing rapidly. High risk EC patients and advanced EC patients often have tumor metastasis and recurrence. The high-risk factors leading to the recurrence of EC include advanced stage, high grade, deep myometrial invasion, obesity and other factors [2, 3]. The five-year survival rate for early EC is 90%, while the five-year survival rate for advanced EC is only 20% [4].

To explore the early detection of diagnostic markers and prognostic markers in EC patients can effectively improve the prognosis. In previous studies, it was found that HE4, CA125, FGF 21 and relative telomere length (RTL) in cell-free DNA (cfDNA) have important value in early diagnosis of EC [5, 6]. On the other hand, the exploration of some preoperative biomarkers can also provide a good idea for the precise treatment and postoperative care of endometrial cancer, for example, preoperative leukocytosis may be associated with poor prognosis of endometrial carcinoma [7], and predictive Score of Nodal Involvement can be used for accurate treatment of endometrial carcinoma by avoiding unnecessary lymph node resection [8].

However, there is still a lack of economic and effective methods to predict the prognosis of EC. Exploring more simple and effective preoperative prognostic indicators will help to develop personalized treatment plans for high-risk patients, thus improving the prognosis.

Serum ferritin (SF) is a complex formed by deferritin and iron core Fe^3+^. It is an indicator of iron storage in the body [9], which can be used to determine whether there is iron deficiency or high iron content in the body. In recent years, SF has been increasingly recognized as a new tumor marker, such as pancreas cancer and colon cancer [10, 11]. And studies have shown that SF is also related to high-risk factors for tumor recurrence such as lung carcinoma [12]. Some research has found that the reason for poor prognosis of tumors may be due to the ferroptosis mechanism [13, 14]. Through the ferroptosis mechanism, the iron can participate in harmful free radical formation reactions. Including Fenton reaction, in which the chain reaction between divalent iron ion (Fe^2+^) and hydrogen peroxide catalyzes the formation of hydroxyl radical. This reaction will not only destroy lipids and proteins, but also cause oxidative damage to DNA [15, 16]. There is no relevant report on serum ferritin as a prognostic index in EC. Therefore, the purpose of this study was to explore the role and value of SF level in predicting the prognosis of patients with EC.

## Material and methods

### Patients

We retrospectively analyzed 367 patients diagnosed with EC in our hospital from January 2012 to August 2018. All cases were confirmed by pathology, including histological types of adenocarcinoma, serous carcinoma, clear cell carcinoma and so on. Our staging was based on FIGO (Federation International of Gynecology and Obstetrics) 2009 system [17]. Clinicopathological data (Age, FIGO stage, grade, myometrial invasion and lymph node metastasis, information related to serum ferritin) were obtained from the patient's medical records, serum ferritin was measured by Electrochemiluminescence. The criteria for including patients in our study are as follows: 1. Patients diagnosed with EC. 2. Patients whose serum ferritin was tested before surgery. 3. Patients with hematological diseases were excluded. Their average age is 55 years old, and the age range is 32–78 years old. All patients were grouped according to tumor grade (G1, G2, G3) and depth of myometrial invasion (< 0.5, ≥ 0.5). All patients were divided into endometrioid carcinoma and non-endometrioid carcinoma according to the histological subtype.

### Statistical analysis

We used SPSS software for statistical analysis. All continuous variables were showed by mean ± standard deviation (SD). The independent sample t-test was used to compare the continuous variables which follow the normal distribution. ROC curve (receiver operating characteristic) was used to evaluate the sensitivity and specificity of SF in patients with EC. The optimal cut-off value of SF was determined by maximizing the Youden index (Sensitivity + Specificity-1). Hosmer–lemeshow test was used to confirm the goodness of fit of the model. Categorical variables were compared using the chi-squared test and presented as numbers with percentage values. Kaplan–Meier method and log-rank were used to analyze OS and DFS of high serum ferritin group and low serum ferritin group. Schoenfeld's residuals test was used to evaluate Cox proportional hazards model. Cox regression analysis was used to evaluate the independent prognostic factors of EC. OS was calculated in months between the date of diagnosis and the date of death or last follow-up, while DFS was calculated in months between the end of the primary cancer treatment and the date of recurrence or death/last follow-up. Recurrent disease was defined as a histopathologically or radiologically documented disease occurred. When *P* < 0.05, we considered it statistically significant.

## Results

### Histopathological findings

According to inclusion and exclusion criteria, we retrospectively analyzed 367 patients diagnosed with EC in our hospital from January 2012 to August 2018. Among 367 patients with EC, the specific data are shown in Table 1. The median follow-up time was 61 months (interquartile range [IQR], 46–76 months). Among them, 59 patients relapsed and 41 patients died of the disease.

**Table 1:** Patient demographics and main pathological features of the group of endometrioid endometrial carcinoma patients (*n* = 367)

Variable	Group	N	%
Age(years)	Mean(± SD)	55.45(± 9.87)	
BMI(kg/m^2^)	Mean(± SD)	24.61(± 3.70)	
Stage			
	I	252	68.66
	II	55	14.99
	III	57	15.53
	IV	3	0.82
Type	1	239	65.12
	2	128	34.88
Histology	Endometrioid carcinoma grade 1	70	19.07
	Endometrioid carcinoma grade 2	169	17.71
	Endometrioid carcinoma grade 3	65	17.71
	Serous carcinoma	37	10.08
	Clear cell carcinoma	11	3.00
	Carcinosarcoma	3	0.82
	Mixed carcinoma	12	3.27
Myometrial invasion	< 50%	247	67.30
	≥ 50%	120	32.70
Lymph node	Absent	346	94.28
Metastasis	Present	21	5.72
Recurrence			
	Yes	58	15.80
	No	309	84.20

*FIGO* International Federation of Gynecology and Obstetrics

### Diagnostic value of SF in different groups

**Fig. 1:**
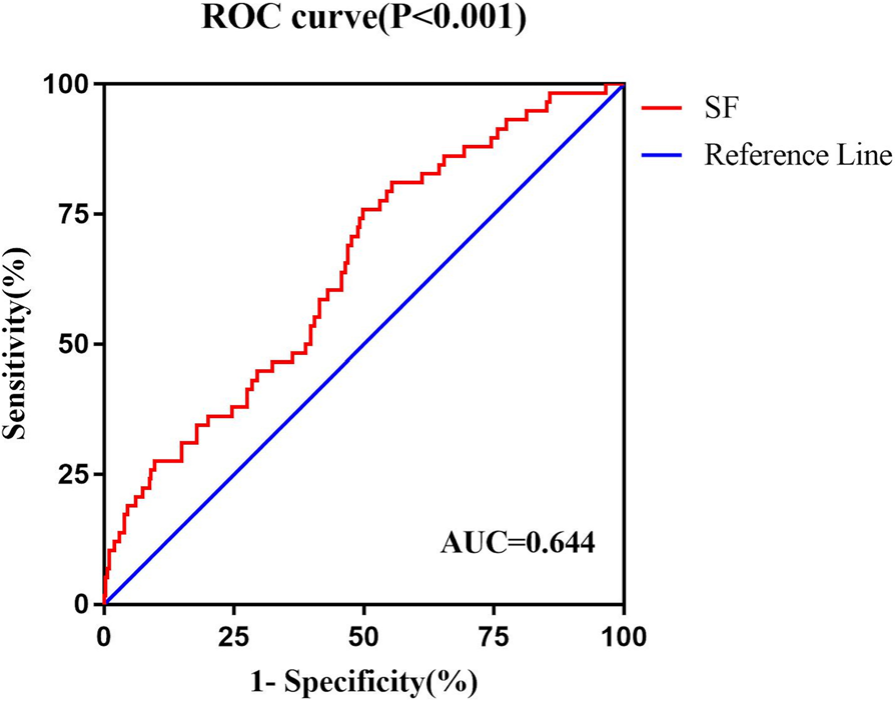
The receiver operating characteristic curves of SF

Area under curve (AUC) determines the cut-off value as well as sensitivity and specificity (Fig. 1). ROC curve analysis maximized the Youden index (Sensitivity + Specificity-1) of the two endpoints of DFS, and determined that 84.27(ng/ml) was the most discriminating cut-off value for SF (95%CI:0.644(0.569–0.719), *P* < 0.001, Table 2). To confirm the model's goodness of fit, a Hosmer–Lemeshow test was performed, which could not reveal bad calibration (*P* = 0.103).

**Table 2:** Diagnostic performance of assessment of SF

Group/	Cut-off value	Sensitivity	Specificity	Accuracy	AUC(95% CI)	*P* value
Parameter	ng/ml	%	%	%		
SF	84.27	75.9	50.2	63.1	0.644(0.569–0.719)	< 0.001

*SF *Serum ferritin, *AUC *Area under the ROC curve, *CI *Confidence interval, *ROC *Receiver operating characteristic

### Comparison of SF values among different groups

According to the cut-off value of SF, SF ≥ 84.27 was defined as SF high expression group, and SF < 84.27 was defined as SF low expression group. The high expression group of SF was significantly correlated with the recurrence of EC (HR 3.163,95%CI:1.666–6.008, *P* < 0.001). In addition, it was also related to older age (*P* = 0.002), specific histological subtype (HR:2.204,95%CI:1.411–3.443, *P* < 0.001) and high grade (*P* = 0.010, Table 3).

**Table 3:** Clinicopathological characteristic by SF

Variable (*N* = 367)	SF ≤ 84.27 ng/ml (*N* = 169)	SF > 84.27 ng/ml (*N* = 198)	*P*
Age(years)			
Mean(± SD)	52.91 ± 10.12	57.62 ± 9.16	< 0.001
BMI(kg/m^2^)			
Mean(± SD)	24.39 ± 4.07	24.80 ± 3.36	0.108
Type			< 0.001
I	126	113	
II	43	85	
Histology			< 0.001
Endometrioid carcinoma	153	151	
Non-endometrioid carcinoma	16	47	
Grade			0.010
G1	46	24	
G2	80	89	
G3	27	38	
Stage			0.254
I			
II			
III			
IV	1	2	
Myometrial invasion			0.065
< 50%	122	125	
≥ 50%	47	73	
Lymph node			
Metastasis			0.762
Absent	160	186	
Present	9	12	
Recurrence			< 0.001
Yes	14	44	
No	155	44	

### SF predicts prognosis in patients: results of survival analysis

**Fig. 2:**
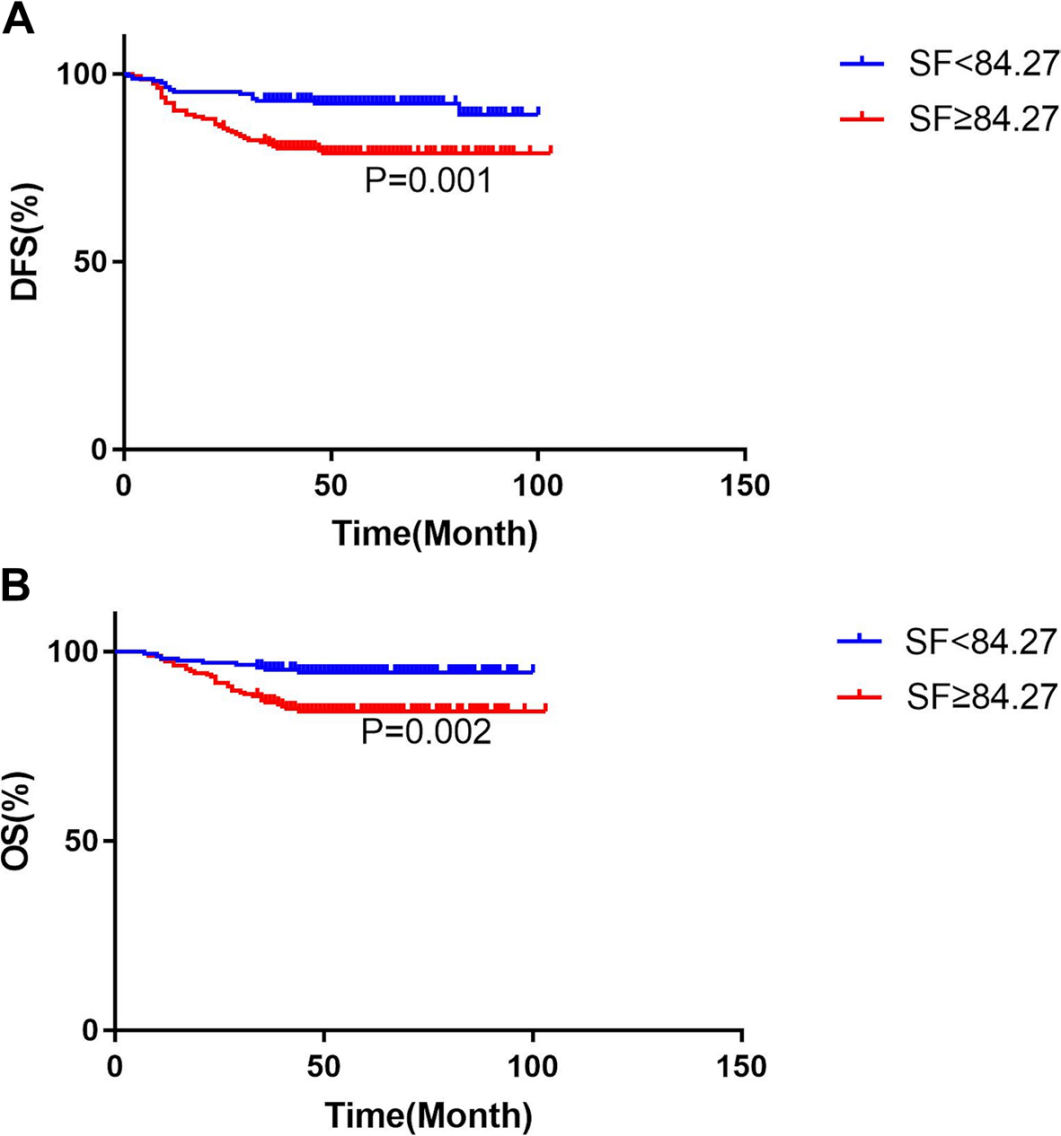
Kaplan–Meier curves for the disease-free survival (**a**) and overall survival (**b**) of 367 patients, according to SF cut-off values were 84.27 ng/ml

A total of 367 patients were included in the present study. The mean SF of patients with recurrence was 217.346 ng/ml, which was higher than that of patients without recurrence (119.267 ng/ml). The mean SF of dead patients was 206.579 ng/ml, which was higher than that of alive patients (125.736 ng/ml). The DFS and OS of 198 patients with elevated preoperative serum ferritin levels were significantly lower than those with low serum ferritin levels (HR 2.670, 95%CI:1.453–4.909, *P* = 0.001 and HR 3.044, 95%CI:1.445–6.412, *P* = 0.002, respectively, Fig. 2). The univariate cox analysis was used to analyze the clinicopathological factors may affect the prognosis of EC (Table 4). Multivariate analysis using the Cox regression model showed that SF level were independent prognostic indicators for DFS (HR 2.290, 95%CI:1.195–4.385, *P* = 0.012) and OS (HR 2.294, 95% CI:1.101–5.650, *P* = 0.028). The results are presented in Table 5.

**Table 4:** Univariate prognostic analysis

Factors	HR	DFS 95% CI	*P*	HR	OS 95% CI	*P*
SF						
≤ 84.27	1.0			1.0		
>84.27	2.670	1.453-4.909	0.002	3.044	1.445-6.412	0.003
Age						
≤ 55.45	1.0			1.0		
> 55.45	2.129	1.199-3.782	0.010	3.332	1.582-7.018	0.002
Type						
I	1.0			1.0		
II	3.332	1.927–5.763	< 0.001	6.109	2.977–12.539	< 0.001
Histology						
Endometrioid	1.0					
Non-endometrioid	3.914	2.272–6.743	< 0.001	4.831	2.572–9.073	< 0.001
Stage						< 0.001
I	1.0			1.0		
II	1.731	0.728–4.11	0.215	0.996	0.286–3.465	0.995
III	9.238	5.122–16.662	< 0.001	8.976	4.555–17.687	< 0.001
IV	11.124	1.483–83.451	0.019	8.690	1.141–66.181	0.037
myometrial invasion						
< 50%	1.0			1.0		
≥ 50%	2.937	1.716–5.025	< 0.001	2.625	1.398–4.927	0.003
Lymph node						
Metastasis						
Absent	1.0					
Present	3.981	1.876–8.450	< 0.001	2.998	1.170–7.681	0.022

All unknown data and uncommon histology were not included in the analysis. Abbreviations: CI, confidence interval; HR, hazard ratio; DFS, Disease-free survival

**Table 5:** Multivariate prognostic analysis

Factors	HR	DFS 95% CI	*P*	HR	OS 95% CI	*P*
SF						
≤84.27	1.0			1.0		
>84.27	2.290	1.195-4.385	0.012	2.494	1.101-5.650	0.028
Age						
≤55.45	1.0			1.0		
>55.45	1.324	0.705-2.488	0.382	1.987	0.893-4.421	0.092
Type						
I	1.0			1.0		
II	1.566	0.740-3.313	0.241	3.798	1.581-9.127	0.003
Histology						
Endometrioid	1.0		1.0			
Non-endometrioid	1.251	0.576-2.717	0.572	0.913	0.393-2.118	0.832
Stage						
I	1.0			1.0		
II	1.991	0.824-4.811	0.119	1.318	0.370-4.688	0.670
III	8.030	3.932-16.398	<0.001	8.963	3.871-20.756	<0.001
IV	10.419	1.208-89.899	0.033	7.638	0.880-66.339	0.065
myometrial invasion						
<50%	1.0			1.0		
≥50%	1.081	0.561-2.086	0.993	0.687	0.313-1.505	0.348
Lymph node						
Metastasis						
Absent	1.0			1.0		
Present	1.0	0.278-1.674	0.404	0.632	0.632	0.401

*Abbreviations: CI* Confidence interval, *HR* Hazard ratio

## Discussion

Our clinical studies have shown that high SF levels are associated with poor prognosis of EC, the high expression of SF level was also positively correlated with the high-risk factors leading to the recurrence of EC (older age, specific histological subtype, high grade).

EC occurs in endometrial epithelium, most of which are adenocarcinoma. It is a common cancer in the female reproductive system. It is more common in women aged 50 ~ 60 years old. EC has a good overall prognosis, but once recurrence and distant metastasis, the survival rate will be greatly reduced. In recent years, the incidence rate and mortality rate of EC are increasing [18], and the incidence population is gradually younger. Considering the need of fertility of young patients, personalized treatment of EC is very important. The fertility-sparing and ovarian preservation options depend on the histological type, depth of myometrial invasion, grade and stage of EC. Therefore, it is very important to accurately evaluate the stage and grade of EC and other high-risk factors before operation. However, the treatment of EC mainly depends on the postoperative pathological results. At present, there is no effective method to evaluate these factors before operation. Peripheral blood indicators have non-invasive and economic advantages over other indicators. Therefore, we analyzed the peripheral blood clinical data of 367 patients with EC in our hospital and found that SF was an independent prognostic factor for DFS and OS of EC (*P* = 0.012, *P* = 0.028). In our cohort study, according to the maximization of Youden index, the optimal cut-off value of SF is 84.27 ng/ml. We also refer to the iron overload standard stipulated by WHO (World Health Organization) and divide SF into two groups. Low SF was defined as ≤ 150 ng/ml and high SF was defined as > 150 ng/ml in the female population [19]. In patients with EC, DFS and OS in the high SF expression group were still significantly lower than those in the low serum ferritin expression group (Figure S1, Figure S2). However, we do not know whether the iron overload index of Asian women deviates from the index specified by the WHO, so we adopted the maximization of the Youden index as the cut-off value. Therefore, SF may be used as a biomarker to evaluate the high-risk factors of EC, predict the prognosis, and guide the personalized treatment of EC. In addition, patients after surgery should also be treated according to whether there are high-risk factors. Patients with high-risk factors have a high recurrence rate. Radiotherapy or systemic treatment should be supplemented and follow-up should be strengthened to reduce the recurrence rate of patients. In our study, it was found that high levels of SF are associated with non-endometrioid carcinoma (*P* = 0.010), and histological types have guiding significance for the selection of EC surgical methods. For example, ovarian preservation may be safe, for stage I endometrioid carcinoma [20, 21]. Therefore, SF may be used as a specific biomarker for preoperative evaluation of high-risk factors and postoperative personalized treatment of patients with EC, so as to improve the prognosis of EC.

Serum ferritin is usually considered as the best clinical index of human iron reserve. However, higher concentration of serum ferritin is related to the development of some diseases. Previous studies have demonstrated that high SF expression is closely related to the poor prognosis of ovarian cancer, pancreatic cancer, liver cancer and other diseases [22–24]. This is consistent with our conclusion. The increase in SF is closely related to the poor prognosis of patients with EC: The DFS and OS of 198 patients with elevated serum ferritin levels were significantly lower than those with low serum ferritin levels (*P* = 0.001 and* P* = 0.002, respectively).

We speculate that the mechanism of poor prognosis of endometrial carcinoma caused by high level of serum ferritin is mainly related to insulin resistance, infiltration of inflammatory factors and ferroptosis. JM Fernandez-Real et al. believe that serum ferritin may be a marker of insulin resistance [25]. This may be because iron deposition in the liver will interfere with the function of insulin to inhibit hepatic glucose production, resulting in insulin resistance. Christine M Friedenreich et al. included 514 patients with EC and 962 controls. It was found that the increase of insulin was associated with the increased risk of EC, indicating that insulin resistance is a potential risk factor of EC [26, 27]. In conclusion, elevated SF may play a role in the poor prognosis of EC through the mechanism of insulin resistance.

The occurrence and development of tumor are also related to inflammation and oxidative stress. The continuous infiltration of inflammatory factors is a favorable condition for tumor, and inflammatory factors interact with oxidative stress factors to form a tumor microenvironment conducive to tumor development [28]. In the nineteenth century, Vichow put forward the concepts of cancer and inflammation. Inflammatory cells in tumors are more likely to promote tumor growth, progression and immunosuppression [29]. Serum ferritin can not only be used as an indicator of inflammatory response, but also increase oxidative stress through Fenton reaction. It has been reported that the increase of SF can promote the infiltration of inflammatory factors in the tumor microenvironment [30, 31]. Studies have shown that women’s serum ferritin levels are related to inflammatory index C-reactive protein (CRP) and Body Mass Index (BMI) [31]. In EC, inflammatory factors can also promote the development of EC by increasing the expression of estrogen [32, 33], moreover, another factor associated with SF, BMI, is not only a poor prognostic factor of EC, but also can induce the inflammatory environment of the body, thus forming a favorable microenvironment for tumor [34, 35]. Therefore, SF may lead to poor prognosis of EC by affecting the infiltration of inflammatory factors in EC. In summary, studying the specific mechanism of SF may become a new target for EC therapy.

We also speculate that the poor prognosis of EC caused by SF may be related to the ferroptosis mechanism. The ferroptosis was first reported by Dr. Stocker in 2008. It is an iron-dependent non-apoptotic form that can regulate the process of cell death [36]. Iron is necessary for cell replication, metabolism and growth [37]. However, iron can also participate in harmful free radical formation reactions. Including Fenton reaction, in which the chain reaction between divalent iron ion (Fe^2+^) and hydrogen peroxide catalyzes the formation of hydroxyl radical. This reaction will not only destroy lipids and proteins, but also cause oxidative damage to DNA [15, 16]_._ More and more studies have found that ferroptosis is closely related to the growth regulation of various tumor cells such as ovarian cancer, liver cancer, and pancreatic cancer [38–40]. Therefore, iron is both necessary and potentially toxic. A variety of related drugs have been found to affect the occurrence of ferroptosis in tumor cells. For example, recent studies have shown that artesunate can selectively induce ferroptosis in pancreatic ductal adenocarcinoma cell lines with mutational activity [41]. However, our research on the signaling pathways and transcription factors involved in the mechanism of ferroptosis is still in a vague stage. Further study on the mechanism of ferroptosis can provide more possible therapeutic targets for clinical application, and it is expected to develop new drugs and therapeutic methods for these targets.

In our study, the 84.27(ng/ml) cut-off of SF was used to predict OS in patients with EC. Based on this threshold, univariate analysis showed that SF was associated with poor prognosis. According to multivariate analysis, SF level were independent prognostic indicators for DFS and OS. Our results verify the good prognostic value of SF in EC patients. However, our study has some limitations. (1) There is not enough inflammatory index data to analyze whether the poor prognosis of EC caused by elevated SF is related to the inflammatory mechanism of tumor. (2) This is a single center retrospective study, so our findings need to be verified in large-scale prospective studies. (3) The specific mechanism of ferroptosis leading to poor prognosis of EC is not clear, and further research is needed. (4) Our research lacks of patients' history of iron supplement consumption. There are many studies showing the significant effect of iron supplements on the increased level of Ferritin [42, 43].

## Conclusions

Our study found that serum ferritin is an indicator of poor prognosis of EC and is positively correlated with high-risk factors of EC recurrence. The detection of serum ferritin is noninvasive and economical, so it may be a good index to evaluate the prognosis of patients with endometrial cancer.

### Supplementary Information

**Additional file 1.**

**Additional file 2.**

**Additional file 3.**

**Additional file 4.**

**Additional file 5.**

## Data Availability

Raw data were obtained in the supplementary material.
