# Supplementary figures and images for: The crucial value of serum ferritin in assessing high-risk factors and prognosis for patients with endometrial carcinoma
Source: BMC Womens Health. 2023 Aug 7;23:415. doi: 10.1186/s12905-023-02575-x (PMC10408112; doi:10.1186/s12905-023-02575-x)

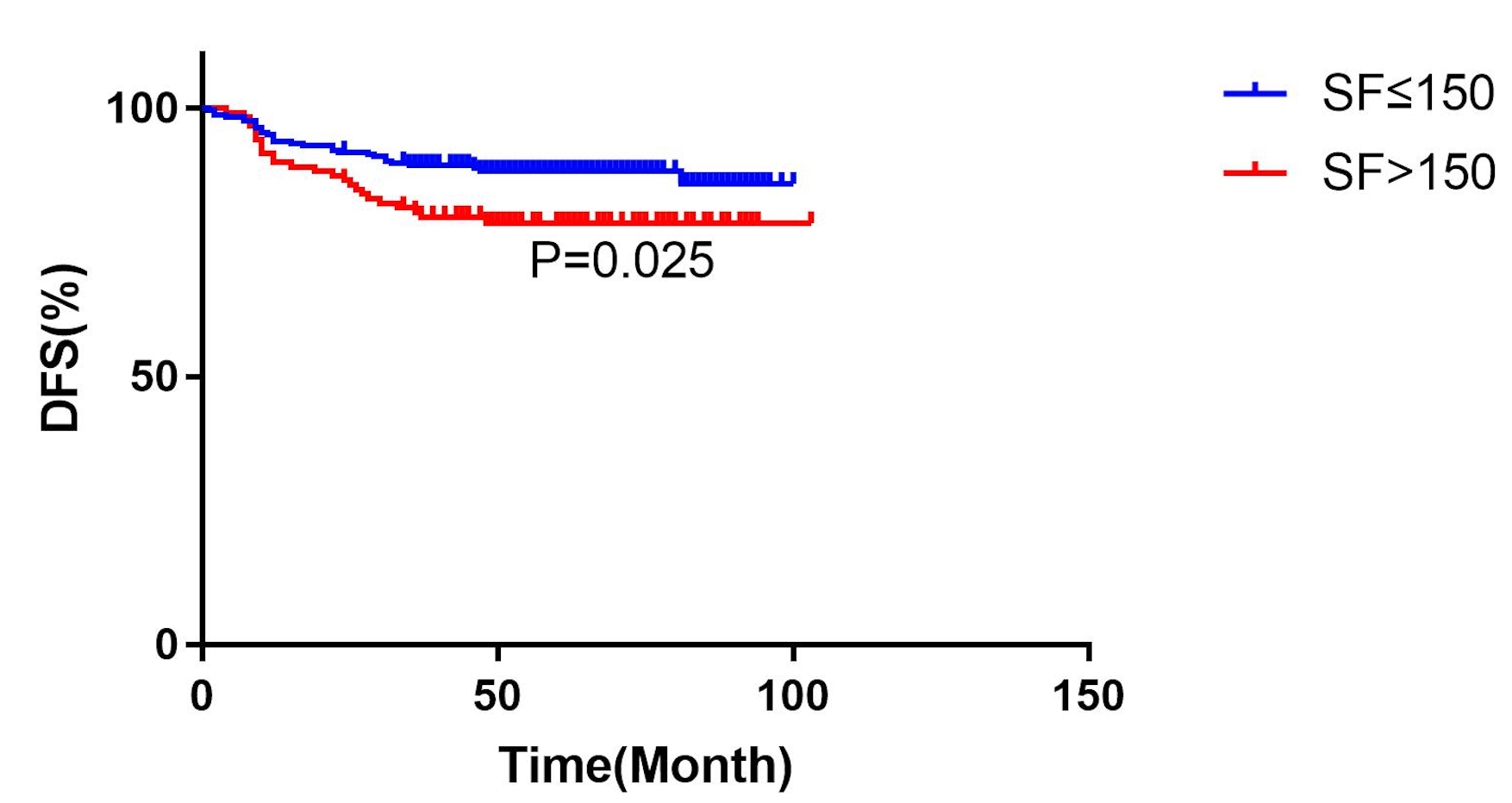

Supplement: Supplementary file 1 — Additional file 1. [file 12905_2023_2575_MOESM1_ESM.jpg]

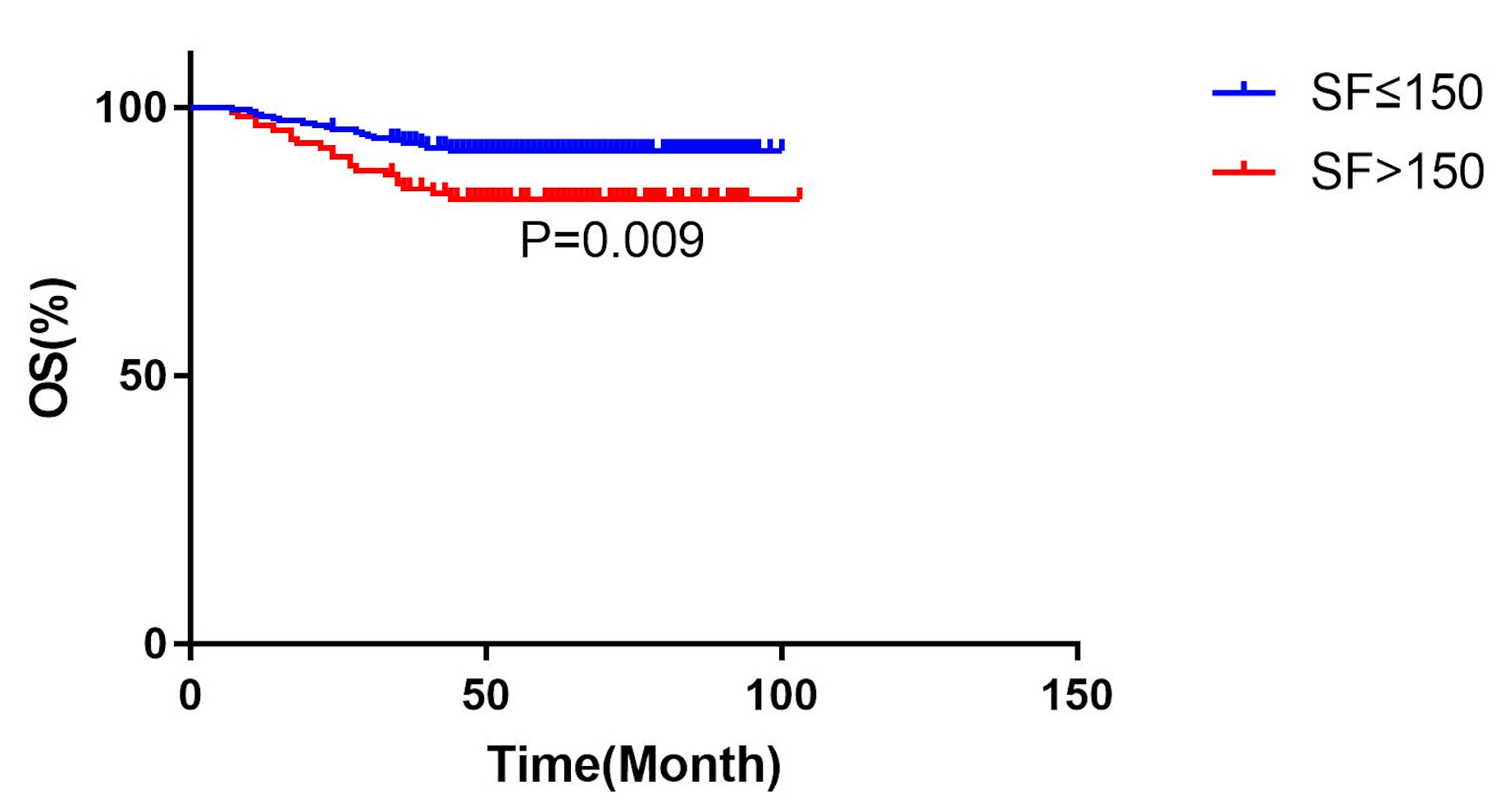

Supplement: Supplementary file 2 — Additional file 2. [file 12905_2023_2575_MOESM2_ESM.jpg]
