# Supplementary material for: The crucial value of serum ferritin in assessing high-risk factors and prognosis for patients with endometrial carcinoma
Source: BMC Womens Health. 2023 Aug 7;23:415. doi: 10.1186/s12905-023-02575-x (PMC10408112; doi:10.1186/s12905-023-02575-x)
Supplement: Supplementary file 3 — Additional file 3. [file 12905_2023_2575_MOESM3_ESM.docx]

SF row Data 2: The original data of this study: including the patient's age, weight, height, FIGO stage, grade，myometrial invasion and lymph node metastasis, recurrence and death of patients information related to serum ferritin. This original data was from the First Affiliated Hospital of Chongqing Medical University, and has passed the informed consent and ethical approval of patients.
