# Supplementary material for: The crucial value of serum ferritin in assessing high-risk factors and prognosis for patients with endometrial carcinoma
Source: BMC Womens Health. 2023 Aug 7;23:415. doi: 10.1186/s12905-023-02575-x (PMC10408112; doi:10.1186/s12905-023-02575-x)
Supplement: Supplementary file 4 — Additional file 4. [file 12905_2023_2575_MOESM4_ESM.docx]

Figure. S1 Kaplan–Meier curves for the disease-free survival of 367 patients, according to SF cut-off values were 150 ng/ml (According to WHO). HR 1.828, 95%CI:1.071-3.122, *P=*0.025. SPSS software was used to statistical analysis.

Figure. S2 Kaplan–Meier curves for the overall survival of 367 patients, according to SF cut-off values were 150 ng/ml (According to WHO). HR 2.247 95%CI:1.199-4.210, *P*=0.009. SPSS software was used to statistical analysis.
